# Adrenal Ganglioneuroblastoma of Adult Onset: A Literature Review

**DOI:** 10.7759/cureus.33288

**Published:** 2023-01-03

**Authors:** Christopher M Stevens, Kevin Malone, Kylie Dufrene, William Mclean, Prerana Ramesh, Norris Talbot, Amro Saad Aldine, Octavio Arevalo

**Affiliations:** 1 Interventional Radiology, Louisiana State University Health Sciences Center, Shreveport, USA; 2 Biomedical Engineering, Louisiana State University Health Sciences Center, Shreveport, USA; 3 Radiology, Louisiana State University Health Sciences Center, Shreveport, USA; 4 Interventional Radiology, University of Texas Southwestern Medical Center, Dallas, USA; 5 Neuroradiology, Louisiana State University Health Sciences Center, Shreveport, USA

**Keywords:** age and sex, tumors, adrenal disease, adult onset, ganglioneuroblastoma

## Abstract

Ganglioneuroblastomas (GNBs) are a rare subtype of neoplastic tumors that arise from the autonomic nervous system and contain both mature gangliocytes and immature neuroblasts. The primary age group affected by GNBs is the pediatric population, with less than 50 cases of adult GNBs existing in the literature. To the authors’ best knowledge, only 21 cases of GNBs arising in the adrenal glands of adults have been reported. Herein we present a literature review examining the symptoms, treatment type, age, and sex of adults, and the presence of tumor metastases and calcification from the 21 cases reported in the literature.

## Introduction and background

Neuroblastic tumors arise from primitive cells of the sympathetic nervous system and are composed of three histological subtypes: neuroblastoma, ganglioneuroblastoma (GNB), and ganglioneuroma [1,2]. These three tumors vary in terms of malignancy, with neuroblastomas being the most malignant of the three and the most common extracranial solid tumor in childhood [3,4]. Ganglioneuromas are categorized as benign tumors while GNBs express an intermediate malignant potential [3]. Neuroblastic tumors can arise anywhere within the sympathetic nervous system although most occur in the adrenal glands [1]. Cases in adolescence and adulthood are extremely rare but have been reported in the literature [1,5-7].

GNBs contain both mature gangliocytes and immature neuroblasts and are composed of two subgroups, nodular-GNBs (stroma poor) and intermixed-GNBs (stroma rich) [1]. The majority of GNB cases arise by 10 years of age and have a median diagnosis of 22 months, with less than 50 cases of GNBs in adults being reported in the literature [8,9]. To the authors’ best knowledge, only 21 cases of GNBs arising in the adrenal glands of adults have been reported. Herein, we present a literature review examining the symptoms, treatment type, age and sex of the adult, and the presence of tumor metastases and calcification from these 21 cases.

## Review

The inclusion criteria for our study consisted of all cases in the literature that reported an adrenal GNB in an adult (>18 years of age). Searching PubMed and Google Scholar for articles of this type yielded 21 cases. A literature review, examining the symptoms, treatment type, age and sex of the patients, and presence of tumor metastases and calcification from these 21 cases, was conducted (Table 1).

**Table 1:** Results of literature review that examined the symptoms, treatment type, and age and sex of the patients, and the presence of tumor metastases and calcification from the 21 cases in the literature that reported an adrenal GNB of adult-onset. (-) = not reported in case GNB: ganglioneuroblastoma

Authorship and Year of Publication	Symptoms	Treatment	Age (years) and Biological Sex	Metastases	Presence of Calcification
Bolzacchini et al., 2015 [1]	■ Asymptomatic	Surgical removal	63-year-old male	Absent	(-)
Vassalo et al., 2021 [9]	■ Recurrent epigastric pain ■ Diarrhea	Surgical removal	22-year-old male	Absent	Yes
Fujiwara et al., 2000 [10]	■ Recurrent Headaches ■ Nausea ■ Vomiting ■ Hypertension	Surgical removal	25-year-old female	Absent	Yes
Mizuno et al., 2010 [11]	■ Increased frequency of urination	Surgical removal	53-year-old male	Bone	No
Benedini et al., 2017 [12]	■ Flank pain	Surgical removal	20-year-old female	Lymph nodes	Yes
Slapa et al., 2002 [13]	■ Asymptomatic	Surgical removal	20-year-old female	Absent	Yes
Heidari et al., 2018 [14]	■ Abdominal discomfort ■ Dysuria ■ Mild hematuria	Surgical removal	38-year-old male	Absent	(-)
Qiu et al., 2015 [15]	■ Swelling and pain of waist and abdomen ■ Fever	Surgical removal	27-year-old female	Absent	Yes
Kumata et al., 2018 [16]	■ Asymptomatic with prior history of hypertension	Surgical removal	73-year-old female	Absent	(-)
Koike et al., 2003 [17]	■ Asymptomatic	Surgical removal	50-year-old male	Absent	No
Hiroshige et al., 1995 [18]	■ Asymptomatic	Surgical removal	35-year-old male	Absent	Yes
Rousseau et al., 1998 [19]	(-)	Surgical removal combined with radiotherapy and chemotherapy	Female, unable to obtain age*	Liver	(-)
Gunlusoy et al., 2004 [20]	■ Flank Pain ■ Malaise ■ Epigastric pain ■ Anemia ■ Weight loss ■ Microscopic hematuria	Surgical removal	59-year-old male	Lymph nodes	(-)
Lonie et al., 2019 [21]	■ Acute iliac fossa pain	Surgical removal	27-year-old male	Absent	(-)
Koizumi et al., 1992 [22]	■ General fatigue and low back pain	None	47-year-old female	Bone marrow	(-)
Sorrentino et al., 2014 [23]	■ Persistent abdominal pain	Surgical removal	28-year-old male	Lymph nodes	(-)
Ding et al., 2015 [24]	■ Loin pain	Surgical removal	27-year-old female	Absent	(-)
Takahashi et al., 1988 [25]	■ Asymptomatic	Surgical removal combined with radiotherapy and chemotherapy	21-year-old male	Lymph nodes	(-)
Cameron et al., 1967 [26]	■ Diarrhea ■ Hypokalaernic nephropathy ■ Thin bones	Surgical removal	54-year-old female	Absent	(-)
Butz, 1940 [27]	(-)	(-)	25-year-old male	Liver; para-aortic nodes	(-)
Higuchi, 1993 [28]	(-)	Surgical removal	29-year-old male	Bone marrow	(-)

Of the 21 cases in the literature review, 12 cases involved males and nine cases were female. The age group most affected was 20-29-year-olds, with 11 of the cases involving patients within this range. Only two cases had patients older than the age of 60, one being male and the other female. The average age of onset was approximately 37.2 years old, with the average age in the male patients being 37.5 years old and female 36.7 years old, excluding the case where age was undetermined. Patients were asymptomatic in six of the cases, in which the tumor was found incidentally upon imaging. Abdominal pain/discomfort, flank pain, epigastric pain, loin pain, low back pain, hematuria, fatigue, dysuria, diarrhea, polyuria, recurrent headaches, vomiting, hypertension, and nausea were all symptoms reported; abdominal pain/discomfort was the most common reported symptom. Being that nearly a third of patients were asymptomatic, the prevalence of adrenal GNBs in adults is likely higher in the general population than what is reported in the literature. Hypertension was reported in two cases, and excision of the tumor corrected hypertension in both cases [10,16]. Surgical removal of GNBs is the most common therapeutic approach as it was performed in 19 of the 21 cases. Additionally, of the two cases that did not receive any form of curative treatment, one did not receive chemotherapy and died three months after initial imaging of identified the adrenal mass [22]. Two cases reported surgical removal combined with radiotherapy and chemotherapy, with one case reporting the use of systemic vincristine and cyclophosphamide therapy along with OK-432 and irradiation of the operated space [25]. Metastases were present in nine of the cases, six males and six females, and undetermined in one case, with a prevalence at the time of diagnosis being 42.9%. Five out of the nine patients with metastases were between the ages of 20-29-year-old population, and the prevalence of metastases in the 20-29-year-old population was 45.5%, which is greater than the overall prevalence in this study. The locations of metastases included the liver, lymph nodes, bone/bone marrow, and para-aortic nodes. Lymph node metastases were the most common, occurring in four out of the nine cases with metastases. Calcification was present in six and undetermined in 13 of the cases. Most, five out of the six, cases with calcification were seen in the 20-29-year-old population and only seen in one case with the presence of metastases, specifically of the lymph node. No calcification was noted in patients 35 years or older or was otherwise not commented on.

Our results from the literature review, including patient age, treatment type, and symptoms, are compatible with the conclusions made from previous literature reviews conducted on the adult onset of adrenal GNB cases [1,9,12]. However, these literature reviews concluded that adrenal GNBs of adult-onset were seen in males with a greater magnitude than our findings. The tumor occurred in 43% of females and 57% of males in our present review but in 24% of females and 76% of males [1], 36.4% of females and 63.6% of males [9], and 37.5% of females, and 62.5% of males [12] in the three previous literature reviews. We believe that these differences are due to errors made by the authors of the previous literature reviews. We recently showed that inaccurate information was presented in the literature review conducted by Bolzacchini et al. [1], causing a misleading conclusion being made about which sex the tumor is seen most in [29]. We also found a mistake in table 3 of the literature review conducted by Benedini et al. [12] when they referenced an adrenal GNB case of adult-onset by Fujiwara et al. Benedini et al. lists the patient from this case [10] as a male when they were a female. In the literature review conducted by Vassallo et al. [9], they say 22 cases of adrenal GNB of adult-onset were reported in the literature and that 14 occurred in males; however, they did not cite all 22 cases. As a result, we were unable to validate their findings. With the extreme rarity of this tumor type, these minor errors made in at least two of the previous literature reviews impacted the statistics of this tumor, thus leading to slightly different conclusions being made about the sex distribution of the tumor compared to the present review. Thus, our results show that while adult-onset adrenal GNBs may affect males at a slightly higher rate than females, the magnitude at which this occurs is probably not as high as what is concluded in the current literature, due to errors being made in previous literature reviews that resulted in misleading conclusions being made, and may even be statistically insignificant due to the small number of cases reported in the literature.

Since GNBs can present with a wide variety of symptoms or asymptomatically, imaging techniques, such as computed tomography (CT), ultrasound, and magnetic resonance imaging (MRI), are of great importance when trying to diagnose GNBs. These imaging modalities can help characterize the mass and are useful in pretreatment risk stratification [9,30]. CT findings of GNBs can vary from a relatively solid mass to a cystic-like mass containing a few thin strands of solid tissue [9]. With MRI, GNBs are usually heterogeneous and of low signal intensity on T1 - weighted images but normally contain higher signal intensity on T2 - weighted images [9]. While these imaging techniques can serve a fundamental role in helping localize and visualize a possible GNB when present, a definitive diagnosis cannot be made until a histopathological analysis is performed due to the great similarity the three neuroblastic tumors display on imaging. According to the International Neuroblastoma Pathology Committee, GNBs are classified into two groups, GNB intermixed (stroma rich) and GNB nodular (stroma poor) [2]. GNB intermixed is characterized by the presence of intermixing neuroblastic and ganglion cells while GNB nodular expresses a stroma poor/neuroblastic nodular component that is normally hemorrhagic/necrotic and coexisting with a stroma rich or stroma dominant component [9].

Regardless of age or tumor location, it is important to keep examining the patient periodically after treatment of GNB, due to the chance of recurrence which mostly occurs within the first two years after surgery [9,31]. Examining the patient every three months for the first two years, then every six months after two years has been reported as good practice [1,9,31]. A thorough examination should involve obtaining a complete blood count, urinary catecholamine synthesis analysis, and imaging of the site where the tumor was first diagnosed [1]. High levels of urinary catecholamine synthesis have been shown to correlate with recurrence [32], thus why it is recommended to evaluate this marker during the patient’s checkups.

## Conclusions

GNBs are a rare subtype of neoplastic tumors that contain both mature gangliocytes and immature neuroblasts. Less than 50 cases of adult GNBs exist in the literature, with only 21 cases of GNBs arising in the adrenal glands of adults having been reported. A literature review, examining the symptoms, treatment type, age, and sex of the patients from the 21 cases of adult-onset adrenal GNB in the literature was conducted. From the review, it was concluded that adrenal GNBs of adult-onset affect males at a slightly higher rate than females, but not to the magnitude that is reported in the current literature by previous literature reviews on this topic, are diagnosed more in the 20-29-year-old patient population than any other adult population, can present with a varying degree of symptoms, are mostly treated by surgical removal of the tumor, and sometimes express metastatic characteristics and calcification.
